# Resequencing and Association Analysis of Six PSD-95-Related Genes as Possible Susceptibility Genes for Schizophrenia and Autism Spectrum Disorders

**DOI:** 10.1038/srep27491

**Published:** 2016-06-07

**Authors:** Jingrui Xing, Hiroki Kimura, Chenyao Wang, Kanako Ishizuka, Itaru Kushima, Yuko Arioka, Akira Yoshimi, Yukako Nakamura, Tomoko Shiino, Tomoko Oya-Ito, Yuto Takasaki, Yota Uno, Takashi Okada, Tetsuya Iidaka, Branko Aleksic, Daisuke Mori, Norio Ozaki

**Affiliations:** 1 Department of Psychiatry, Nagoya University Graduate School of Medicine, 466-8550 Nagoya, Japan; 2 Institute for Advanced Research, Nagoya University, 466-8550 Nagoya, Japan

## Abstract

PSD-95 associated PSD proteins play a critical role in regulating the density and activity of glutamate receptors. Numerous previous studies have shown an association between the genes that encode these proteins and schizophrenia (SZ) and autism spectrum disorders (ASD), which share a substantial portion of genetic risks. We sequenced the protein-encoding regions of DLG1, DLG2, DLG4, DLGAP1, DLGAP2, and SynGAP in 562 cases (370 SZ and 192 ASD patients) on the Ion PGM platform. We detected 26 rare (minor allele frequency <1%), non-synonymous mutations, and conducted silico functional analysis and pedigree analysis when possible. Three variants, G344R in DLG1, G241S in DLG4, and R604C in DLGAP2, were selected for association analysis in an independent sample set of 1315 SZ patients, 382 ASD patients, and 1793 healthy controls. Neither DLG4-G241S nor DLGAP2-R604C was detected in any samples in case or control sets, whereas one additional SZ patient was found that carried DLG1-G344R. Our results suggest that rare missense mutations in the candidate PSD genes may increase susceptibility to SZ and/or ASD. These findings may strengthen the theory that rare, non-synonymous variants confer substantial genetic risks for these disorders.

Schizophrenia (SZ) is a debilitating disorder that affects approximately 1% of the population. A large portion of its heritability, which is estimated at 80%^1^, remains to be explained. Results from multiple large-scale genome-wide association studies as well as whole-genome/whole-exome sequencing support a polygenic model for explaining the susceptibility to the disorder. In this model, deleterious rare variants exert significantly larger effects than common single nucleotide polymorphisms (SNPs)^2^,^3^,^4^.

The postsynaptic density (PSD) is a protein complex localized at the postsynaptic plasma membrane of excitatory synapses. The PSD is essential for protein trafficking in neurons and synaptic plasticity^5^, processes commonly associated with the pathogenesis of SZ^6^. Scaffolding proteins, the primary components of the PSD structure, interact closely with glutamate receptors and play a major role in the dynamic regulation of their signaling activities^7^. PSD-95, which is a key protein in this subgroup, is a member of the membrane-associated guanylate kinase (MAGUK) family and is encoded by the disks large homolog 4 (*DLG4*) gene. Its linkage to SZ has been well established through both variant association^8^ and expression studies^9^,^10^,^11^,^12^,^13^,^14^. *DLG1* and *DLG2*, which encode two other MAGUK family proteins, synapse-associated protein 97 (SAP97) and postsynaptic density protein 93 (PSD-93), respectively, have been similarly linked to SZ^9^,^15^,^16^,^17^,^18^,^19^,^20^. The products of *DLGAP1* and *DLGAP2* are guanylate kinase-associated protein (GKAP) family proteins that bind to the MAGUKs, mediating their interaction with other components of the PSD complex^21^,^22^. Resequencing studies have implicated these genes as susceptibility genes for SZ^23^,^24^. SynGAP1, another major scaffolding protein, has multiple protein-protein interacting motifs that enable it to act as a structural and regulatory anchor in synaptic homeostasis^6^. Its association with SZ has been shown in human expression studies and animal models^10^,^25^,^26^.

Recently, many whole-genome/whole-exome sequencing studies focusing on deleterious rare mutations, including copy number variants (CNVs), have frequently identified the PSD gene group, especially the PSD-95-related subgroup. In a study conducted by Purcell *et al.* who analyzed the exome sequences of 2536 SZ cases and 2543 controls for the burden of rare, disruptive mutations, the PSD, activity-regulated cytoskeleton-associated scaffolding protein, and PSD-95 gene sets were associated with SZ (p = 0.0808, p = 0.0016, p = 0.0017, for singletons, respectively)^2^. Multiple *de novo* CNVs spanning the coding regions of *DLG1*, *DLG2*, and *DLGAP1* have been discovered in European and Asian SZ patients^27^. A Swedish study of 4719 SZ cases and 5917 controls found a significantly increased burden of large CNVs (>500 kb) in genes present in the PSD, especially in the 3q29/*DLG1* locus, which has been implicated in previously conducted genome-wide association studies^28^.

Autism spectrum disorders (ASD) are a range of conditions characterized by persistent deficits in social communication and interaction, as well as restricted, repetitive patterns of behavior, interests, or activities. Both ASD and SZ belong to a group of distinct clinical entities known as neurodevelopmental disorders, as defined in DSM-V^29^. It has been indicated by clinical and epidemiologic studies that neurodevelopmental disorders have a high comorbidity rate, overlapping signs and symptoms, and significant similarities in genetic background^30^,^31^,^32^. Furthermore, various previous researches provide strong evidences of common underlying molecular pathways and shared genetic causes between ASD and SZ^4^,^33^,^34^,^35^,^36^,^37^. A recent review of targeted large-scale resequencing studies has pointed out that genetic evidence converges on three functional pathways, one of which is synaptic function. This review also predicted that PSD genes such as *DLG4* and *SynGAP1* will be identified as key nodes in the connected network^38^. A similar study utilizing the network-based analysis of genetic associations system identified a large biological network of genes that are affected by rare *de novo* CNVs in autism, with *DLG4*, *DLG1*, and *DLG2* as important nodes in the cluster^39^. Individually, *DLGAP2* and *SynGAP1* are established risk genes for ASD^40^,^41^,^42^,^43^,^44^,^45^, and *DLG1*, *DLG2*, and *DLG4* have also been implicated in various studies^46^,^47^,^48^,^49^.

Based on the results of these studies, we selected six candidate genes with the most evidence implicating an association with SZ and ASD: *DLG1*, *DLG2*, *DLG4*, *DLGAP1*, *DLGAP2*, and *SynGAP1*. The exonic regions of these genes were sequenced to look for rare, protein-altering point mutations.

## Materials and Methods

### Participants

Two independent sample sets were used in this study (Table 1). The first set, comprising 370 SZ patients (mean age = 49.73 ± 14.75 years; males = 52.97%) and 192 ASD patients (mean age = 16.34 ± 8.36 years; males = 77.60%), was sequenced for rare point mutations. The second, larger set, comprising 1315 SZ patients (mean age = 47.41 ± 15.35 years; males = 53.92%), 382 ASD patients (mean age = 19.61 ± 10.71 years; males = 77.75%), and 1793 controls (mean age = 45.11 ± 14.61 years; males = 51.25%), was used for association analysis of selected variants detected in the first set.

All participants in this study were recruited in the Nagoya University Hospital and its associated Institutes. Patients were included in the study if they (1) met DSM-5 criteria for SZ or ASD and (2) were physically healthy. Controls were selected from the general population and had no personal or family history of psychiatric disorders (first-degree relatives only based on the subject’s interview). The selection was based on the following: (1) questionnaire responses from the subjects themselves during the sample inclusion step; or (2) an unstructured diagnostic interview conducted by an experienced psychiatrist during the blood collection step. All subjects were unrelated, lived in the central area of the Honshu island of Japan, and self-identified as members of the Japanese population. The Ethics Committees of the Nagoya University Graduate School of Medicine approved this study. All experiments were performed in accordance with the Committee’s guidelines and regulations. Written informed consent was obtained from all participants. In addition, each patient’s capacity to provide consent was confirmed by a family member when needed. Individuals with a legal measure of reduced capacity were excluded.

### Resequencing and Data Analysis

Genomic DNA was extracted from whole blood or saliva using the QIAGEN QIAamp DNA blood kit or tissue kit (QIAGEN Ltd., Germany). Custom amplification primers were designed to cover coding exons and flanking intron regions of the selected genes with Ion AmpliSeq Designer (Thermo Fisher Scientific, USA). Sample amplification and equalization were achieved using Ion AmpliSeq Library Kits 2.0 and the Ion Library Equalizer Kit, respectively (Thermo Fisher Scientific, USA). Amplified sequences were ligated with Ion Xpress Barcode Adapters (Thermo Fisher Scientific, USA). Emulsion PCR and subsequent enrichment were performed using the Ion OneTouch Template Kit v2.0 on Ion OneTouch 2 and Ion OneTouch ES, respectively (Thermo Fisher Scientific, USA). The final product was then sequenced on the Ion PGM sequencing platform (Thermo Fisher Scientific, USA). Raw data output from the sequencer was deposited in the DNA Data Bank of Japan (DDBJ) (http://www.ddbj.nig.ac.jp) under the accession number DRA004490, and uploaded to the Torrent Server (Life Technologies, USA) for variant calling, with NCBI GRCh37 as a reference. The resulting VCF files were analyzed by Ingenuity Variant Analysis (QIAGEN Ltd., Germany) for annotation and visualization.

### Association Analysis

Missense mutations, small insertions/deletions, and splicing site variations with a minor allele frequency <1% were selected from the annotated data. The mutation calls were then validated for confidence by Sanger sequencing using the BigDye Terminator v3.1 Cycle Sequencing Kit (Applied Biosystems, USA). Genotyping prioritization was based on whether the mutation was 1) located in a functional domain or motif of the protein, according to the Human Protein Reference Database (http://www.hprd.org), Pfam (http://pfam.xfam.org/), and existing literature^21^,^50^,^51^,^52^,^53^,^54^,^55^,^56^,^57^,^58^,^59^, 2) functionally important, such as causing a frame shift, stop gain, or cysteine gain/loss, 3) novel, as in not documented in the NCBI dbSNP database (Build 137) (http://www.ncbi.nlm.nih.gov/SNP/), the 1000 Genomes Project (http://www.1000genomes.org/), the Exome Variant Server of NHLBI GO Exome Sequencing Project (ESP6500SI-V2) (http://evs.gs.washington.edu/EVS/), or the Human Genetic Variation Database of Japanese genetic variation consortium (http://www.genome.med.kyoto-u.ac.jp/SnpDB), and 4) predicted to be deleterious by *in silico* analytic methods. In addition to PolyPhen-2 (http://genetics.bwh.harvard.edu/pph2/) and SIFT (http://sift.jcvi.org/) that were originally incorporated in the Ingenuity Variant Caller, we also employed PROVEAN (http://provean.jcvi.org/index.php), PMut (http://www.ngrl.org.uk/Manchester/page/pmut), Mutation Taster (http://www.mutationtaster.org/), and PANTHER (http://pantherdb.org/) for enhanced prediction of the consequences of protein alterations.

Custom TaqMan SNP genotyping assays were designed and ordered from Applied Biosystems. Allelic discrimination analysis was performed on an ABI PRISM 7900HT Sequence Detection System (Applied Biosystems, USA). Differences in allele and genotype frequencies of the mutations were compared between SZ patients/controls and ASD patients/controls using Fisher’s exact test (two-tailed), with a threshold of significance set at p < 0.05.

### Additional Analysis for Amino Acid Changes

Conservation status of genotyping candidates in 11 common species was investigated using HomoloGene (http://www.ncbi.nlm.nih.gov/homologene). Possible 3D changes caused by mutations in the protein structure were predicted and modelled with I-TASSER (http://zhanglab.ccmb.med.umich.edu/I-TASSER/) and UCSF Chimera (http://www.cgl.ucsf.edu/chimera/).

## Results

### Resequencing and Genetic Association Analyses

**Figure 1:**
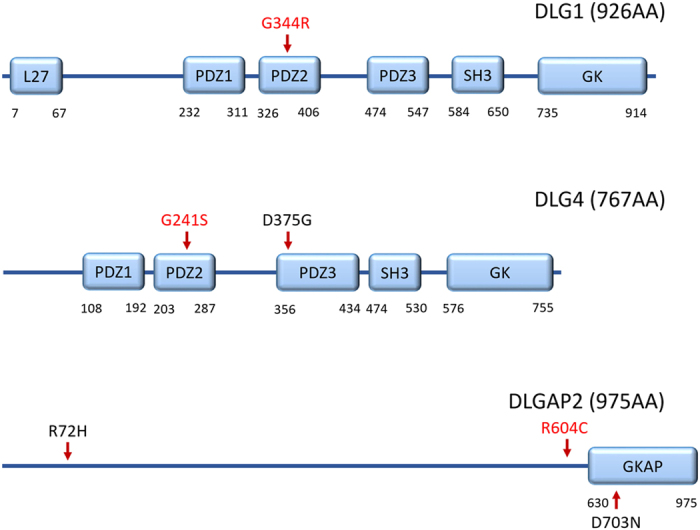
Locations of amino acid changes caused by detected mutations in the *DLG1*, *DLG4* and *DLGAP2* genes. 1. Protein sequence and domain data was obtained from Human Protein Reference Database. 2. Mutations validated in association analysis are marked in red.

Thirty-seven rare, non-synonymous mutations were called by Ingenuity Variant Analysis during resequencing. Among them, 26 were validated via the Sanger method (Table 2). All variants were heterozygous. The carriers of four variants had pedigree DNA available. Sanger sequencing revealed that all four were inherited. Based on the selection criteria mentioned in Materials and Methods, G344R in *DLG1*, G241S in *DLG4*, and R604C in *DLGAP2* were selected for association analysis (Fig. 1). The *DLG4*-G241S and *DLGAP2*-R604C variants were not found in any of the samples used for genotyping, whereas an additional *DLG1*-G344R variant carrier was detected in the SZ sample group (Table 3).

### Protein 3D Structure Analysis

**Figure 2:**
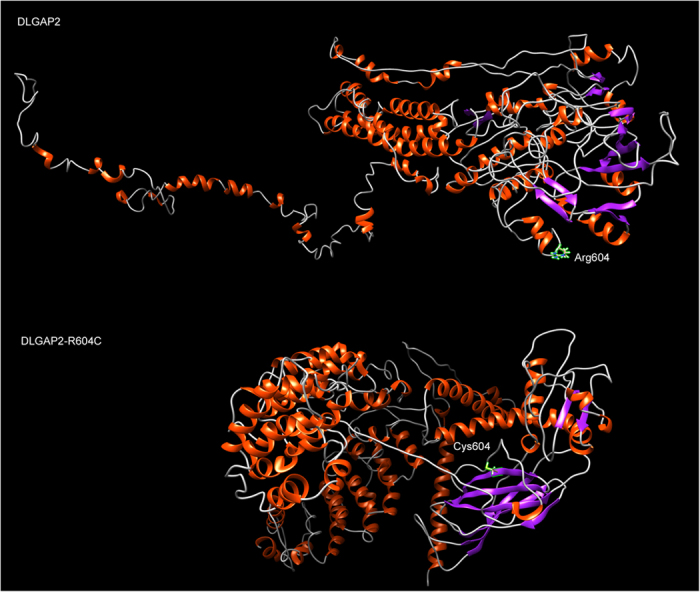
Predicted protein structure of mutated DLGAP2 protein with the R604C variant compared to the wildtype. α-helixes are marked in red and β-strands in purple.

3D modeling of the wild-type and mutated protein sequences indicated that for the *DLGAP2*-R604C variant, the additional cysteine gained from the mutation significantly changes the secondary and tertiary structures by adding a local β strand (Fig. 2).

### Evolutionary Conservation Analysis

Results obtained from HomoloGene showed that the amino acids corresponding to the three mutations in *DLG1*, *DLG4*, and *DLGAP2* were highly conserved among different species (Table S1).

### Clinical Information of Mutation Carriers

Detailed descriptions of the clinical information can be found in the Supplement. Interestingly, the variant *DLGAP2*-R604C in one ASD patient was inherited from a parent who is also affected with ASD.

## Discussion

Both SZ and ASD are disorders involving polygenic inheritance, with rare variants having a much higher impact on susceptibility than common variants. Recent large-scale genetic studies have reported that ultra-rare and private non-synonymous mutations are highly enriched in patient populations, especially in sets of genes with functions closely involved in neurodevelopment^2^,^60^,^61^,^62^,^63^. *DLG4*-G241S and *DLGAP2*-R604C were only present in single cases among a collective sample size of 562 patients during resequencing as well as in 1697 patients and 1793 controls, and *DLG1*-G344R was present in two SZ cases from the same sample sets. Therefore, they may confer a much higher risk than regular rare mutations discovered with the criterion of a minor allele frequency of <1%.

The second PDZ domain (PDZ2) of SAP-97, where *DLG1*-G344R is located, folds into a compact globular domain comprising six β-strands and two α-helices, which is a typical architecture for PDZ domains. During synaptic transmission, SAP-97 interacts with key protein partners such as ligand-binding units in α-amino-3-hydroxy-5-methyl-4-isoxazolepropionic acid receptor (AMPAR)^53^,^64^ and N-methyl-D-aspartate receptor (NMDAR)^57^ through the PDZ2 domain to regulate glutamate signaling and neuronal growth, which are major factors in the pathogenesis of SZ and ASD. The same domain also functions as a binding site for receptors for the neurotrophic growth factors corticotropin-releasing hormone (CRF)^65^ and epidermal growth factor receptor (ErbB1)^66^, which are linked to SZ.

The first PDZ domain (PDZ1) of PSD-95, where *DLG4*-G241S is located, similarly binds to NMDAR^57^. PDZ1 is also the site at which PSD-95 interacts with other PSD proteins such as SynGAP^67^. Mutated PDZ domains have been linked to defective PSD clustering and dendrite spine morphology in cultured cells^67^, as well as disrupted glutamate signaling and learning ability in animal models^68^. Interestingly, one study showed an association of this domain with Angelman Syndrome, a genetic disorder exhibiting a high occurrence rate in patients with autism, due to its functional relevance in the TrkB-PSD-95 signaling pathway^69^.

Cysteine is a ‘special’ amino acid that forms disulfide bonds between cysteine residues. These bonds are the basis of secondary and quaternary structures and are critical for the stabilization of tertiary structures of a protein^70^,^71^. The presence of *DLGAP2*-R604C introduces a new cysteine to the protein sequence and is highly likely to cause the formation or breaking of a disulfide bond that in turn disrupts the normal folding of DLGAP2.

The Exome Aggregation Consortium (ExAC, http://exac.broadinstitute.org/) integrates the exome sequencing data from 60,706 unrelated individuals from various studies and populations, which was reprocessed through the same pipeline, and jointly variant-called. While individuals in this dataset aren’t necessarily healthy controls since they only removed subjects with pediatric diseases, it is a useful reference set of allele frequencies due to its scale and data consistency. We searched ExAC for the frequencies of variants we detected in our study (Table S2). It should be noted that *DLG1*-G344R and *DLG4*-G241S did not exist in the database, while *DLGAP2*-R604C was found twice in European (non-Finnish) subjects.

Several limitations should be considered when interpreting the results of our study. First, our relatively small sample set did not have sufficient power to detect statistical significance in an association analysis^72^. Second, we did not conduct molecular biological analysis of the detected mutations. The *in vitro* and *in vivo* impacts of these mutations on the pathophysiology of the disorders need to be examined in future research. In addition, our stringent criteria for selection of variants for further analyses may have left out potentially interesting targets, such as *DLG4*-D375G, which is located in the PDZ domain of the encoded protein and was predicted by four *in silico* tools to be pathological. In addition, R72H and D703N in *DLGAP2* are not present in a known functional domain but were predicted to be pathological by all five tools. These variants may be good candidates for a follow-up study (Fig. 1). Finally, our sequencing did not cover the promoter, untranslated regions, or intronic regions of the target genes, which may contain important mutations at regulatory sites.

## Conclusion

In this study, we sequenced the exonic regions of PSD-95 and related genes in SZ and ASD patients using the Ion PGM platform and discovered 26 rare, non-synonymous variants. We then conducted an association analysis in a much larger sample set for three of these variants to investigate their relationship with SZ and/or ASD. Although statistical significance was not obtained, the observation that these mutations were only detected in cases, together with the structural relevance and *in silico* prediction results, indicates that they may impact the susceptibility of carriers to these disorders.

## Additional Information

**How to cite this article**: Xing, J. *et al.* Resequencing and Association Analysis of Six PSD-95-Related Genes as Possible Susceptibility Genes for Schizophrenia and Autism Spectrum Disorders. *Sci. Rep.* **6**, 27491; doi: 10.1038/srep27491 (2016).

## Supplementary Material

Supplementary Information

## Figures and Tables

**Table 1:** Profiles of participants in the resequencing and association sample sets.

	Resequencing		Association analysis		Controls
	SZ	ASD	SZ	ASD	
Total	370	192	1315	382	1793
Males	196 (52.97%)	149 (77.60%)	709 (53.92%)	297 (77.75%)	919 (51.25%)
Females	174 (47.03%)	43 (22.40%)	606 (46.08%)	85 (22.25%)	874 (48.75%)
Mean age (years)	49.73 ± 14.75	16.34 ± 8.36	47.41 ± 15.35	19.61 ± 10.71	45.11 ± 14.61

**Table 2:** Rare, non-synonymous mutations identified during the resequencing stage.

Genomic Position	Gene Symbol	Transcript Variant	Protein Variant	Case Samples With Variant	SIFT Prediction	PolyPhen-2 Prediction	Mutation Taster Prediction	PROVEAN Prediction	PANTHER Prediction	dbSNP ID	1000 Genomes Frequency	HGVD Frequency	Domain	Pedigree Analysis
3:196786778	*DLG1*	c.2186A>T	p.K855I	1	Damaging	Benign	Disease Causing	Deleterious	Deleterious			0.001	GK	
3:196812488	*DLG1*	c.1552G>C	p.E634Q	1	Damaging	Probably Damaging	Disease Causing	Neutral	Neutral			0.008	SH3	
3:196812570	*DLG1*	c.1470C>G	p.N606K	1	Damaging	Benign	Disease Causing	Neutral	Neutral				SH3	
3:196857519	*DLG1*	c.1143A>C	p.E381D	1	Tolerated	Benign	Disease Causing	Neutral	Neutral			0.001	PDZ	
3:196863502	*DLG1*	c.1030G>C	p.G344R	1	Damaging	Probably Damaging	Disease Causing	Deleterious	Deleterious				PDZ	
3:197009653	*DLG1*	c.215C>T	p.P72L	1	Tolerated	Benign	Disease Causing	Neutral	Neutral			0.001		Inherited
8:1496995	*DLGAP2*	c.136G>A	p.D46N	1	Damaging	Possibly Damaging	Disease Causing	Neutral	Neutral	58497511				
8:1497230	*DLGAP2*	c.371G>T	p.R124L	1	Damaging	Probably Damaging	Disease Causing	Deleterious	Neutral					Inherited
8:1497379	*DLGAP2*	c.520G>A	p.A174T	1	Tolerated	Benign	Polymorphism	Neutral	Neutral					
8:1574928	*DLGAP2*	c.1225A>G	p.S409G	1	Tolerated	Benign	Polymorphism	Neutral	Neutral			0.001		
8:1574992	*DLGAP2*	c.1289C>T	p.S430F	1	Damaging	Probably Damaging	Disease Causing	Deleterious	Deleterious	201068222	0.02			
8:1616734	*DLGAP2*	c.1810C>T	p.R604C	1	Damaging	Probably Damaging	Disease Causing	Neutral	Deleterious					Inherited
8:1624733	*DLGAP2*	c.1997G>A	p.R652H	1	Damaging	Possibly Damaging	Polymorphism	Neutral	Neutral	375426065	0.04	0.002	GKAP	
8:1626417	*DLGAP2*	c.2044G>A	p.A696T	1	Damaging	Probably Damaging	Polymorphism	Neutral	Neutral			0.003	GKAP	
8:1626550	*DLGAP2*	c.2219C>A	p.T740N	1	Damaging	Probably Damaging	Disease Causing	Deleterious	Deleterious				GKAP	
8:1626657	*DLGAP2*	c.2284G>A	p.V776I	1	Tolerated	Probably Damaging	Disease Causing	Neutral	Neutral			0.001	GKAP	
11:83984282	*DLG2*	c.17T>A	p.V6D	1	Tolerated	Benign	Polymorphism	Neutral	Neutral					
11:84822760	*DLG2*	c.302C>T	p.P101L	1	Tolerated	Probably Damaging	Disease Causing	Neutral	Neutral					
17:7100164	*DLG4*	c.1124A>G	p.D375G	1	Tolerated	Probably Damaging	Disease Causing	Deleterious	Deleterious				PDZ	
17:7106562	*DLG4*	c.583G>A	p.G241S	1	Damaging	Probably Damaging	Disease Causing	Deleterious	Deleterious				PDZ	Inherited
18:3534411	*DLGAP1*	c.2260G>A	p.D754N	1	Tolerated	Possibly Damaging	Disease Causing	Neutral	Neutral	376569562			GKAP	
18:3534564	*DLGAP1*	c.1273G>A	p.D703N	1	Damaging	Probably Damaging	Disease Causing	Deleterious	Deleterious				GKAP	
18:3742510	*DLGAP1*	c.1175T>C	p.I392T	2	Tolerated	Benign	Disease Causing	Neutral	Neutral			0.002		
18:3879572	*DLGAP1*	c.497G>A	p.G166D	3	Tolerated	Benign	Disease Causing	Neutral	Deleterious					
18:3879854	*DLGAP1*	c.215G>A	p.R72H	1	Damaging	Probably Damaging	Disease Causing	Deleterious	Deleterious					
18:3880047	*DLGAP1*	c.22C>A	p.R8S	1	Damaging	Probably Damaging	Disease Causing	Deleterious	Deleterious			0.001		

1. Based on NCBI Build GRCh37/hg19.

2. Positions of allele/amino acid changes are determined with reference to the following RefSeq accessions:

DLG1: NM_004087.2; NP_004078.2

DLGAP2: NM_004745.3; NP_004736.2

DLG2: NM_001142699.1; NP_001136171.1

DLG4: NM_001365.3; NP_001356.1

DLGAP1: NM_004746.2; NP_004737.2

3. GK: guanylate kinase-like domain; SH3: SRC homology 3 domain; PDZ: PSD95-Dlg1-zo1 domain; GKAP: guanylate kinase-associated protein domain.

**Table 3:** Association analysis results of three rare missense mutations

Variant	Genotype counts (resequencing)^a^		Genotype counts (association)			P value	
	SZ	ASD	SZ	ASD	Control	SZ	ASD
*DLG1*-G344R	0/1/739	0/0/384	0/1/2629	0/0/764	0/0/3586	0.4231	–
*DLG4*-G241S	0/0/740	0/1/383	0/0/2630	0/0/764	0/0/3586	–	–
*DLGAP2*-R604C	0/0/740	0/1/383	0/0/2630	0/0/764	0/0/3586	–	–

^a^: Homozygote of minor allele/heterozygote/homozygote of major allele.
