# Supplementary material for: Resequencing and Association Analysis of Six PSD-95-Related Genes as Possible Susceptibility Genes for Schizophrenia and Autism Spectrum Disorders
Source: Sci Rep. 2016 Jun 7;6:27491. doi: 10.1038/srep27491 (PMC4895433; doi:10.1038/srep27491)
Supplement: Supplementary Information [file srep27491-s1.doc]

# Resequencing and Association Analysis of Six PSD-95-Related Genes as Possible Susceptibility Genes for Schizophrenia and Autism Spectrum Disorders (Supplementary Information)

Jingrui Xing^1^, Hiroki Kimura^1^, Chenyao Wang^1^, Kanako Ishizuka^1^, Itaru Kushima^1,2^, Yuko Arioka^1^, Akira Yoshimi^1^, Yukako Nakamura^1^, Tomoko Shiino^1^, Tomoko Oya-Ito^1^, Yuto Takasaki^1^, Yota Uno^1^, Takashi Okada^1^, Tetsuya Iidaka^1^, Branko Aleksic^1^,*, Daisuke Mori^1^, and Norio Ozaki^1^

^1^ Department of Psychiatry, Nagoya University Graduate School of Medicine, 466-8550 Nagoya, Japan

^2^ Institute for Advanced Research, Nagoya University, 466-8550 Nagoya, Japan

^*^ Corresponding author. Address: 466-8550, 65 Tsurumai-cho, Showa-ku, Nagoya, Japan. Tel: +81 52 744 2282. Fax: +81 52 744 2293. E-mail: [branko@med.nagoya-u.ac.jp](mailto:branko@med.nagoya-u.ac.jp)

**Table S1. Evolutionary conservation status for three association analysis candidates.**

| Mutation | Protein Accession Number | Starting AA | Alignment | Ending AA |
| --- | --- | --- | --- | --- |
| *DLG1*-G344R | [NP_004078.2](http://www.ncbi.nlm.nih.gov/entrez/viewer.fcgi?db=protein&id=148539578) | 321 | KLIKGPKGLGFSIAGGVGNQHIP**G**DNSIYVTKIIEGGAAHKDGKLQIGDK | 370 |
|  | [XP_001166204.1](http://www.ncbi.nlm.nih.gov/entrez/viewer.fcgi?db=protein&id=114591294) | 321 | KLIKGPKGLGFSIAGGVGNQHIP**G**DNSIYVTKIIEGGAAHKDGKLQIGDK | 370 |
|  | [XP_001098808.1](http://www.ncbi.nlm.nih.gov/entrez/viewer.fcgi?db=protein&id=109054014) | 321 | KLIKGPKGLGFSIAGGVGNQHIP**G**DNSIYVTKIIEGGAAHKDGKLQIGDK | 370 |
|  | [XP_545159.2](http://www.ncbi.nlm.nih.gov/entrez/viewer.fcgi?db=protein&id=74002970) | 321 | KLIKGPKGLGFSIAGGVGNQHIP**G**DNSIYVTKIIEGGAAHKDGKLQIGDK | 370 |
|  | [XP_002684901.2](http://www.ncbi.nlm.nih.gov/entrez/viewer.fcgi?db=protein&id=528936991) | 321 | KLIKGPKAFTTEEVEIVRNGR-RQDKSSNSQGCLVSKQPRPSGK------ | 363 |
|  | [NP_031888.2](http://www.ncbi.nlm.nih.gov/entrez/viewer.fcgi?db=protein&id=40254642) | 321 | KLIKGPKGLGFSIAGGVGNQHIP**G**DNSIYVTKIIEGGAAHKDGKLQIGDK | 370 |
|  | [NP_036920.1](http://www.ncbi.nlm.nih.gov/entrez/viewer.fcgi?db=protein&id=6978763) | 320 | KLIKGPKGLGFSIAGGVGNQHIP**G**DNSIYVTKIIEGGAAHKDGKLQIGDK | 369 |
|  | [XP_004943373.1](http://www.ncbi.nlm.nih.gov/entrez/viewer.fcgi?db=protein&id=513199448) | 321 | KLVKGPKGLGFSIAGGVGNQHIP**G**DNSIYVTKIIEGGAAHKDGKLQIGDK | 370 |
|  | [NP_955820.1](http://www.ncbi.nlm.nih.gov/entrez/viewer.fcgi?db=protein&id=41054776) | 327 | KLVKGPKGLGFSIAGGVGNQHIP**G**DNSIYITKIIEGGAAHKDGRLQIGDK | 376 |
|  | [NP_996406.1](http://www.ncbi.nlm.nih.gov/entrez/viewer.fcgi?db=protein&id=45554813) | 332 | DLVKGGKGLGFSIAGGIGNQHIP**G**DNGIYVTKLMDGGAAQVDGRLSIGDK | 381 |
|  | [XP_003436951.1](http://www.ncbi.nlm.nih.gov/entrez/viewer.fcgi?db=protein&id=347963472) | 254 | ELLKGSKGLGFSIAGGIGNQHIP**G**DNGIYVTKIMEGGAAHIDGRLAVGDK | 303 |
|  | [NP_001039116.1](http://www.ncbi.nlm.nih.gov/entrez/viewer.fcgi?db=protein&id=113931336) | 320 | KLVKGPKGLGFSIAGGVGNQHIP**G**DNSIYVTKIIEGGAAHKDGRLQIGDK | 369 |
| *DLG4*-G241S | [NP_001356.1](http://www.ncbi.nlm.nih.gov/entrez/viewer.fcgi?db=protein&id=4557529) | 201 | VMEIKLIKGPKGLGFSIAGGVGNQHIPGDNSIYVTKIIEG**G**AAHKDGRLQ | 250 |
|  | [XP_001168837.3](http://www.ncbi.nlm.nih.gov/entrez/viewer.fcgi?db=protein&id=410051008) | 200 | VMEIKLIKGPKGTGFSIAGGVGNQHLSGDNSIYVTKIIEG**G**AAHKDGRLQ | 249 |
|  | [XP_001105556.1](http://www.ncbi.nlm.nih.gov/entrez/viewer.fcgi?db=protein&id=109113036) | 201 | VMEIKLIKGPKGLGFSIAGGVGNQHIPGDNSIYVTKIIEG**G**AAHKDGRLQ | 250 |
|  | [XP_005620016.1](http://www.ncbi.nlm.nih.gov/entrez/viewer.fcgi?db=protein&id=545497902) | 143 | LMEIKLIKGPKGLGFSIAGGVGNQHIPGDNSIYVTKIIEG**G**AAHKDGRLQ | 192 |
|  | [NP_001178236.1](http://www.ncbi.nlm.nih.gov/entrez/viewer.fcgi?db=protein&id=300796829) | 155 | LMEIKLIKGPKGLGFSIAGGVGNQHIPGDNSIYVTKIIEG**G**AAHKDGRLQ | 204 |
|  | [NP_031890.1](http://www.ncbi.nlm.nih.gov/entrez/viewer.fcgi?db=protein&id=6681195) | 158 | IIEIKLIKGPKGLGFSIAGGVGNQHIPGDNSIYVTKIIEG**G**AAHKDGRLQ | 207 |
|  | [NP_062567.1](http://www.ncbi.nlm.nih.gov/entrez/viewer.fcgi?db=protein&id=9665227) | 158 | VMEIKLIKGPKGLGFSIAGGVGNQHIPGDNSIYVTKIIEG**G**AAHKDGRLQ | 207 |
|  | [XP_001340947.5](http://www.ncbi.nlm.nih.gov/entrez/viewer.fcgi?db=protein&id=528481123) | 184 | VTELKLIKGPKGLGFSIAGGVGNQHIPGDNSIYVTKIIEG**G**AAHKDGRLQ | 233 |
| *DLGAP2*-R604C | [NP_001264090.1](http://www.ncbi.nlm.nih.gov/entrez/viewer.fcgi?db=protein&id=461496466) | 579 | -GLYNSTDSLDSNKAMNLALETA----AAQ**R**HLPESQSSSVR-TSDKAIL | 622 |
|  | [XP_519583.3](http://www.ncbi.nlm.nih.gov/entrez/viewer.fcgi?db=protein&id=332825526) | 579 | -GLYNSTDSLDSNKAMNLALETA----AAQ**R**HLPESQSSSVR-TSDKAIL | 622 |
|  | [XP_005640779.1](http://www.ncbi.nlm.nih.gov/entrez/viewer.fcgi?db=protein&id=545556403) | 658 | -GLYNSTDSLDSNKAMSLALETA----AAQ**R**LTSDGQSTSAR-TSDKAVL | 701 |
|  | [XP_005222348.1](http://www.ncbi.nlm.nih.gov/entrez/viewer.fcgi?db=protein&id=529000140) |  | -------------------------------------------------- |  |
|  | [NP_766498.2](http://www.ncbi.nlm.nih.gov/entrez/viewer.fcgi?db=protein&id=225543210) | 662 | GGLYNSMDSLDSNKAMNLALETA----AAQ**R**HAADTQSSSTR-SIDKAVL | 706 |
|  | [NP_446353.2](http://www.ncbi.nlm.nih.gov/entrez/viewer.fcgi?db=protein&id=348041395) | 662 | GGLYNSMDSLDSNKAMNLALESA----AAQ**R**HAADTQSSSTR-SIDKAVL | 706 |
|  | [XP_004940544.1](http://www.ncbi.nlm.nih.gov/entrez/viewer.fcgi?db=protein&id=513178703) | 662 | -GMYNSTDSLDSNKAMNLALETA----AAQ**R**HVSE--SASIR-TSDKAIL | 703 |
|  | [XP_685805.5](http://www.ncbi.nlm.nih.gov/entrez/viewer.fcgi?db=protein&id=528506277) | 660 | -ALYNSTDSLDSAKAVTIAMEAAAMAMAGK**R**HPSTDSHSSVM-TCDKAVL | 707 |
|  | [XP_002935195.2](http://www.ncbi.nlm.nih.gov/entrez/viewer.fcgi?db=protein&id=512840931) | 129 | -GLYNSTDSLDSNKAMNLALESA----AAH**R**HVSEIKSSSVKSTSDKAVL | 173 |

**Notes**:

1. AA: amino acid.
2. AAs corresponding to mutations are shown in bold.
3. NP_004078.2/NP_001356.1/NP_001264090.1: *H. sapiens*; XP_001166204.1/XP_001168837.3/XP_519583.3: *P. troglodytes*; XP_001098808.1/XP_001105556.1: *M. mulatta*; XP_545159.2/XP_005620016.1/XP_005640779.1: *C. lupus*; XP_002684901.2/NP_001178236.1/XP_005222348.1: *B. taurus*; NP_031888.2/NP_031890.1/NP_766498.2: *M. musculus*; NP_036920.1/NP_062567.1/NP_446353.2: *R. norvegicus*; XP_004943373.1/XP_004940544.1: *G. gallus*; NP_955820.1/XP_001340947.5/XP_685805.5: *D. rerio*; NP_996406.1: *D. melanogaster*; XP_003436951.1: *A. gambiae*; NP_001039116.1/XP_002935195.2: *X. tropicalis*.

**Table S2**. Frequencies of detected mutations in the Exome Aggregation Consortium (ExAC) database

| Genomic Position | Gene Symbol | Transcript Variant | Protein Variant | ExAC Frequency |
| --- | --- | --- | --- | --- |
| 3:196786778 | DLG1 | c.2186A>T | p.K855I | Not found |
| 3:196812488 | DLG1 | c.1552G>C | p.E634Q | Not found |
| 3:196812570 | DLG1 | c.1470C>G | p.N606K | 0.00001648 |
| 3:196857519 | DLG1 | c.1143A>C | p.E381D | Not found |
| 3:196863502 | DLG1 | c.1030G>C | p.G344R | Not found |
| 3:197009653 | DLG1 | c.215C>T | p.P72L | Not found |
| 8:1496995 | DLGAP2 | c.136G>A | p.D46N | Not found |
| 8:1497230 | DLGAP2 | c.371G>T | p.R124L | 0.00005286 |
| 8:1497379 | DLGAP2 | c.520G>A | p.A174T | 0.00003095 |
| 8:1574928 | DLGAP2 | c.1225A>G | p.S409G | 0.00001848 |
| 8:1574992 | DLGAP2 | c.1289C>T | p.S430F | 0.00003014 |
| 8:1616734 | DLGAP2 | c.1810C>T | p.R604C | 0.00003764 |
| 8:1624733 | DLGAP2 | c.1997G>A | p.R652H | 0.00001888 |
| 8:1626417 | DLGAP2 | c.2044G>A | p.A696T | Not found |
| 8:1626550 | DLGAP2 | c.2219C>A | p.T740N | Not found |
| 8:1626657 | DLGAP2 | c.2284G>A | p.V776I | Not found |
| 11:83984282 | DLG2 | c.17T>A | p.V6D | Not found |
| 11:84822760 | DLG2 | c.302C>T | p.P101L | Not found |
| 17:7100164 | DLG4 | c.1124A>G | p.D375G | Not found |
| 17:7106562 | DLG4 | c.583G>A | p.G241S | Not found |
| 18:3534411 | DLGAP1 | c.2260G>A | p.D754N | 0.00000825 |
| 18:3534564 | DLGAP1 | c.1273G>A | p.D703N | 0.00001703 |
| 18:3742510 | DLGAP1 | c.1175T>C | p.I392T | Not found |
| 18:3879572 | DLGAP1 | c.497G>A | p.G166D | Not found |
| 18:3879854 | DLGAP1 | c.215G>A | p.R72H | Not found |
| 18:3880047 | DLGAP1 | c.22C>A | p.R8S | 0.00001751 |

Notes:

1. Based on NCBI Build GRCh37/hg19.

2. Positions of allele/amino acid changes are determined with reference to the following RefSeq accessions:

DLG1: NM_004087.2; NP_004078.2

DLGAP2: NM_004745.3; NP_004736.2

DLG2: NM_001142699.1; NP_001136171.1

DLG4: NM_001365.3; NP_001356.1

DLGAP1: NM_004746.2; NP_004737.2

Clinical Information for the Carriers of *DLGAP2*-R604C, *DLG4*-G241S, and *DLG1*-G344R

The carrier of *DLGAP2*-R604C was born to parents both diagnosed with ASD. Her father is Chinese and lived in Hong Kong until the year 1994. At the time of conception of the patient, her father and mother were 43 and 39 years of age, respectively. Due to the age of the mother, the child was delivered by Caesarean section at the end of the 37^th^ week of pregnancy, and weighed 2610 g at birth. She first exhibited a speech developmental delay at around 1.5 years old, but no other developmental problems were noted. She was diagnosed with autism at 2 years old. She was 4 years old at the time of sampling, and she still could not form long sentences and tended to use idiosyncratic expressions, such as shortening longer words into a single syllable. She showed no interest in social interactions, and would rather indulge in solitary activities such as drawing or reading. She would often panic whenever slight changes in her living environment took place and was afraid to take showers. In the Kyoto Scale of Psychological Development 2001 (K-test 2001) administered at the age of her diagnosis, she scored 75 in overall Development Quotient (DQ) and 55 in Postural-Motor (P-M), 83 Cognitive-Adaptive (C-A), and 72 Language-Social (L-S) fields. Her father also carries the mutation, and was diagnosed with autism in 2013. Her mother was diagnosed with Asperger’s Syndrome in 2010.

The carrier of *DLG4*-G241S was a 4-year-old child born at full weight by a normal delivery to healthy, unrelated parents. She was the second child in the family and has a healthy older sister. She first exhibited a speech developmental delay at around 1.5 years old and experienced a setback in speech development (forgetting words she already acquired) at 2 years of age. She showed no attachment towards her parents and did not respond to being called by name. Very limited non-verbal communication was also observed, but no motor skill disruptions or aggressive/repetitive behaviors were noted. She scored an overall DQ of 71 in the K-test and 79, 72, and 63 in the P-M, C-A, and L-S fields, respectively, at the age of 2 years, when she was first diagnosed with autism.

One of the carriers of *DLG1*-G344R in the sequencing group was a female SZ patient who was 58 years of age at the time of sampling. She was born to healthy parents who later gave birth to two younger boys. The carrier had a normal course of development throughout childhood. She started to suffer from hallucinations and illusions of persecution at 22 years. During the course of her illness, her illusions persisted, and she had poor recognition of her illness. In the year 2014, she was warned of elevated blood cholesterol and glucose levels in a health checkup.
